# Signal Transduction in the Footsteps of Goethe and Schiller

**DOI:** 10.1186/1478-811X-7-2

**Published:** 2009-02-04

**Authors:** Karlheinz Friedrich, Jonathan A Lindquist, Frank Entschladen, Edgar Serfling, Gerald Thiel, Arnd Kieser, Klaudia Giehl, Christina Ehrhardt, Stephan M Feller, Oliver Ullrich, Fred Schaper, Ottmar Janssen, Ralf Hass

**Affiliations:** 1 Institute of Biochemistry, University of Jena Medical School, Jena, Germany; 2 Institute of Molecular and Clinical Immunology, Otto von Guericke University, Magdeburg, Germany; 3 Instute of Immunology, Witten/Herdecke University, Witten, Germany; 4 Department of Molecular Pathology, Institute of Pathology, University of Würzburg, Würzburg, Germany; 5 Institute of Medical Biochemistry and Molecular Biology, University of the Saarland, Homburg, Germany; 6 Department of Gene Vectors, Helmholtz Zentrum München – German Research Center for Environmental Health, Munich, Germany; 7 Internal Medicine I, University of Ulm, Ulm, Germany; 8 Institute of Molecular Virology, Westfälische-Wilhelms University, Münster, Germany; 9 Molecular Oncology, Weatherall Institute of Molecular Medicine, University of Oxford, Oxford, UK; 10 Institute of Anatomy, University of Zurich, Zurich, Switzerland; 11 Institute of Biochemistry and Molecular Biology, RWTH Aachen University, Aachen, Germany; 12 Institute of Immunology, University Hospital Schleswig-Holstein, Campus Kiel, Kiel, Germany; 13 Medical University of Hannover, Dept. of Gynecology, Laboratory of Biochemistry and Tumor Biology, Hannover, Germany

## Abstract

The historical town of Weimar in Thuringia, the "green heart of Germany" was the sphere of Goethe and Schiller, the two most famous representatives of German literature's classic era. Not yet entirely as influential as those two cultural icons, the Signal Transduction Society (STS) has nevertheless in the last decade established within the walls of Weimar an annual interdisciplinary Meeting on "Signal Transduction – Receptors, Mediators and Genes", which is well recognized as a most attractive opportunity to exchange results and ideas in the field.

The 12^th ^STS Meeting was held from October 28 to 31 and provided a state-of-the-art overview of various areas of signal transduction research in which progress is fast and discussion lively. This report is intended to share with the readers of CCS some highlights of the Meeting Workshops devoted to specific aspects of signal transduction.

## 

The Workshop on **Transcription Control in Cells and Tissues **was introduced by a Keynote Presentation of **Kerstin Krieglstein **(University of Freiburg) on "TGF-β signalling in nervous development". Upon giving an overview on TGF-β receptor and SMAD factor signalling, the speaker presented experimental evidence on the cross-talk of TGF-β-mediated signalling cascade with other signalling pathways, such as the MAPK/ERK cascade. These and other data suggests that TGF-β not only signals through the SMAD transcription factors but also through a number of other signalling pathways which control such fundamental events as proliferation, differentiation, migration, cell death, angiogenesis and wound healing. This leads often to seemingly opposite experimental outcomes when various cell types have been treated by TGF-β.

Among the four short presentations of the workshop two reports dealt with the synthesis and function of NFAT ('Nuclear Factor of Activated T Cell') factors in lymphoid cells. By analysing the induction conditions of NFATc1 in primary T cells from human blood, **Hanna Bendfeldt **and colleagues (Deutsche Rheumaforschungszentrum, Berlin) came to the conclusion that the activity of p38 protein kinase plays an essential role in the autoregulation of NFATc1 transcription in T cells which is thought to be controlled by NFATc2 and NF-κB. Inhibition of p38 activity leads to suppression of NFATc1/A induction, a short isoform of NFATc1 whose activity supports the survival of peripheral lymphocytes. In contrast, other NFATc proteins, such as NFATc2 and NFATc1/C (a longer NFATc1 isoform) exert a strong pro-apoptotic activity. As discussed by **Friederike Berberich-Siebelt **(University of Würzburg), both NFATc2 and NFATc1/C are sumoylated in their C-termini and, thereby, – as shown for NFATc1/C – are guided to heterochromatic nuclear territories. As a consequence of sumoylation, these NFAT proteins bind to histone deacetylases (HDACs) and can act as suppressors of transcription of interleukin 2 gene and of other genes. This might play a role for gene expression in regulatory T (T_reg_) cells by NFATs which can associate with Foxp3, an important (or the 'master') regulator of T_reg _development and function. By using microarray techniques in studies of human T_reg _cells, **Andrea Tuettenberg **and colleagues (University of Mainz) demonstrated an under-representation of NFAT factors in nuclei of human T_reg _cells. These authors identified Galectin-10 as a T_reg_-specific protein whose expression – as shown by siRNA inhibition – contributes to T_reg_-mediated suppression. As typical for human T_reg _cells, the same authors identified by kinome profiling a NEMO-like protein kinase and a high extent of STAT5b phosphorylation. Similar to NFATs, NF-κB factors play a crucial role in the survival of lymphocytes. **Dirk Mielenz **and colleagues (University of Erlangen) identified Tfg (Trk fused gene) as a partner of Carma-1/Ikk-γ, i.e. of molecules of "canonical" NF-κB signalling cascades. Upon B cell receptor and CD40 triggering, Tfg appears to recruit Carma to the plasma membrane, thereby enhancing the basal and CD40-mediated induced activity of Ikkβ and NF-κB. Tfg is expressed at highest levels in marginal zone and B1 B cells suggesting a particular function in NF-κB activation in these B cell populations.

The Workshop on **Receptor-triggered Pathways **was opened by **Arne Östman **(Karolinska Institute, Stockholm), who presented in his keynote presentation recent achievements in translational science with potential meaning for cancer treatment. Low molecular weight inhibitors of receptor tyrosine kinase activity as well as antibodies blocking the receptors at the extracellular face are widely used and constitute valuable for fighting cancers which rely on constitutive activation or overexpression of such receptors. These treatments, however, are only of limited value when downstream signalling molecules are dysregulated with respect to their activity or expression. New strategies do not only focus on the malignant cells themselves but also on supporting cells expressing growth factors with stimulatory effects on cancer cell proliferation or on angiogenesis. For instance, parakrine signals emanating from cancer associated fibroblasts act as growth factors or as chemokines and, thus, stimulate tumour progression. They may represent novel targets for pharmaceutical intervention.

One of the most prominent regulators of cell survival is the Fas ligand. However, **Maren Paulsen **and colleagues (University of Kiel) demonstrated CD95-dependent accelerated proliferation of TCR/CD3/CD28 activated T-lymphocytes by immobilized agonistic FAS antibodies (reverse signalling). In contrast, neither the soluble protein nor immobilized FasL exert similar activities. FasL rather inhibited the proliferation, indicating a negative reverse signalling capacity of FasL.

Fewer strategies were developed to interfere with downstream signalling events relevant for malignant cell growth. The need to further elucidate signalling mechanisms relevant for the growth control of malignant cells is obvious. This understanding was reflected by several talks. As shown by **Heike Hermanns **(Rudolf Virchow Center Würzburg), chemokine (CCL1,7,8) expression is rapidly induced by Oncostatin M (OSM) in primary human dermal fibroblasts. Expression appears to depend on activation of the MAPK cascade. ERK1/2 affects gene expression whereas p38 affects the half live of CCL mRNA. Although OSM is a strong activator of STAT 1, 3, and 5, STAT activation is not crucial for OSM-initiated CCL expression. Instead, STAT5 activation counteracts CCL1 expression through the induction of the feedback inhibitor CIS.

The proto-oncogene and adaptor protein Gab2 is associated with the signal transduction of many growth factors. This adaptor recruits the VIPs of growth factor signalling, namely Grb2, SHP2, PI-3 kinase and beside other functions contributes to the initiation of the MAPK cascade. **Tilman Brummer **and colleagues (University of New South Wales, Sydney) set up a proteomics-based study to define a phosphorylation map of Gab2, and found new sites phosphorylated by PI-3 kinase. Since these phosphorylated motifs are, on the one hand, recognized by 14.3.3 proteins, and binding of 14.3.3 to Gab2, on the other hand, terminates signalling through Gab2, this mechanism represents a novel negative feedback mechanism for the control of growth factor signalling. Interestingly, this mechanism does not apply for Gab1-mediated signalling.

**Henning Urlaub **and colleagues (University of Göttingen) applied an *in vivo *labelling approach to identify phosphorylation sites in leukocyte adapter protein SLP-65. Detailed kinetic and functional studies revealed a high resolution picture of respective roles by individual phosphorylation sites within the SLP-65 molecule in activation of the MAPK cascade and AP-1-mediated gene regulation.

Treatment of solid tumours, especially of the gastrointestinal track is still one major challenge in cancer therapy. Due to the introduction of new specific inhibitors against receptor tyrosine kinases and signaling molecules such as PI-3-kinases or GTPases of the Ras superfamily, the treatment options have made considerable progress. In the workshop on **Tumour Biology and Cancer Therapy **Götz von Wichert (University of Ulm) focussed his keynote presentation specifically on current concepts and their limitations in the treatment of gastrointestinal tumours. He highlighted several new clinical studies addressing the efficacy of targeting EGF or VEGF receptors. This strategy occurs to be quite logical with regard to the numerous data demonstrating the relevance of EGFR and VEGF signalling in tumour progression. However, several new clinical trials have recently documented significant draw-backs in the implementation of these *in vitro *data to clinical trials. For example patients with colorectal cancer bearing mutations in the *k-ras *gene might even have a poorer prognosis under medication with an EGFR inhibitor. In addition, emerging aspects in the understanding of tumour biology (i.e. circulating tumour cells or tumour stem cells) challenge a number of the established treatment concepts. Thus, only the precise understanding of tumour biology will warrant the development of new treatment strategies, but it will be at the same time one of the most challenging tasks for the future.

In the following short talks, **Susanne Steinert **(University of Jena) presented novel data on the importance of bone morphogenetic protein BMP-2 as a potential prognostic marker for human breast cancer. Expression of BMP-2, which is markedly expressed in small, low-grade breast tumours, was found positively correlated with Bcl-2, several cell cycle regulator proteins and the potential tumour suppressor SFRP-1. Higher BMP-2 levels, particularly in nodal-negative invasive breast carcinoma might, thus, result in a prognostic advantage of these patients.

The impact of the scaffold protein CNK1 on breast carcinoma cell behaviour was addressed by **Rafael D. Fritz **and colleagues (University of Zurich). CNK1 was found to influence the invasion-promoting machinery by interfering with the expression of matrix metalloprotease MT1-MMP via inducible elements and by deregulation of the NF-κB pathway. Silencing of tumour-relevant molecules is becoming more and more relevant in analyzing the functional relevance of individual molecules.

**Britta Eiz-Vesper **and colleagues (Hannover Medical School) applied an RNAi-based approach to permanently silence the expression of the NKG2A/CD94 receptor on NK and T cells. Targeting of NKG2A resulted in enhanced lyses of these cells and might improve the success of cell-based immunotherapeutics.

In the last talk of this session, **Stephan M. Feller **(University of Oxford) discussed new aspects of the structural basis of adaptor proteins Grb2 and Gab2 for oncogenic signalling in tumor cells. Two new atypical SH3 domain interaction sites were discovered in Gab2 which might be crucial for the binding of the C-terminal SH3 (SH3C) domain of Grb2 to Gab2 and, thus, might represent a new target structure for the generation of SH3C domain specific inhibitors.

The Workshop **Signalling Induced By Pathogens **has established itself as a "traditional" constituent of the STS Meetings which readily reflects the increasing general interest in this research field. It is now that we begin to understand why interaction of microbial pathogens with the signal transduction network of their target cells is essential for pathogenesis and biology of infection. Cellular pathways are now being considered as promising targets for the development of anti-pathogenic strategies. This year, presentations on signalling events induced by bacterial infections outnumbered contributions on viral signalling, underscoring our increasing knowledge of how bacteria and their toxins exploit host cell signalling for their own purposes.

In his keynote lecture, **Klaus Aktories **(University of Freiburg) gave an overview on mechanisms by which bacterial toxins modify cellular Rho GTPases. As examples for toxins activating Rho GTPases, the cytotoxic necrotizing factors (CNFs) of *E. coli *and *Yersinia *species were discussed, which deamidate a glutamine residue involved in the switch-off mechanism of the GTPases. Other toxins such as the *Clostridium difficile *toxins A and B inactivate various Rho GTPases by glucosylation. Because Rho GTPases have critical functions in both innate and adaptive immunity, the important role of targeting Rho GTPases for the biology of bacterial infection was discussed.

Most notably, bacterial toxins can also act as mitogens and even induce cell transformation by interacting with cellular signalling. **Katharina F. Kubatzky **and colleagues (University of Heidelberg), demonstrated that the *Pasteurella multocida *toxin (PMT), which causes atrophic rhinitis in pigs, caused anchorage-independent growth of epithelial kidney cells. This finding also points to potential carcinogenic properties of the toxin. Data were presented demonstrating that PMT stimulates the JAK-STAT pathway which was even further enhanced after co-expression of SOCS1. PMT-induced expression of the serine/threonine kinase Pim1 caused phosphorylation of SOCS1 and, thus, accumulation of JAK2 and enhanced signaling which might contribute to the transforming capacity of the bacterial toxin.

**Norbert Reiling **and colleagues (Research Center Borstel) found a novel inverse relationship of the Toll-like receptor/NF-κB and the Wnt/β-catenin pathways after aerosol infection of mice with *Mycobacterium tuberculosis*, which may be involved in preserving cell homeostasis during bacteria-induced inflammation. Initially, Reiling's group had observed that components of the Wnt/Frizzled pathway are differentially expressed after infection with mycobacteria, especially Frizzled1 being induced on macrophages in response to *M. tuberculosis*. During their further studies, they found that Wnt signaling is involved in restoring TLR/NF-κB-induced downregulation of β-catenin.

Another highlight of the session was the presentation by **Sina Bartfeld **(Max-Planck Institute of Infection Biology, Berlin). Bartfeld and collegues established an RNA-interference-based high-throughput assay to identify novel components of the NF-κB pathway. The assay is based on a human epithelial cell line stably expressing a p65-GFP fusion protein whose nuclear translocation can be detected by automated microscopy. An siRNA library was cotransfected to screen for proteins required for p65-GFP translocation in response to various stimuli. Applying this assay they could identify two proteins that were critical for NF-κB activation in response to *Helicobacter pylori *but not TNF-α or IL1-β. One of the two factors, an E3-ligase, had not been linked to the NF-κB pathway before and is now under intense investigation.

Viral proteins that interact with host cellular signalling were also investigated further. **Eike R. Hrincius **(University of Münster) presented data demonstrating that the non-structural protein 1 (NS1) of Influenza A virus binds to the cellular adaptor proteins Crk and CrkL, tereby determining the sensitivity of influenza viruses to the expression of the two adapter proteins. Crk and CrkL exclusively bind to NS1, if the second SH3 motif of NS1 harbours the protein-sequence PPLPPK, which predominantly occurs in NS1 of highly virulent avian influenza virus strains. Binding of NS1 to Crk or CrkL contributes to the suppression of the antiviral JNK/ATF2 pathway. It was also shown that virus strains which are able to bind Crk or CrkL are attenuated upon knockdown of the adapter proteins. Because NS1 is known as a virulence factor whose sequence variation determines pathogenicity of the virus, the specific interaction of NS1 variants with cellular adapter proteins is of great interest to understand the molecular basis for the severity of highly virulent influenza infections.

In the Workshop on **Differentiation, Senescence and Cell Death, Peter Herrlich **(Leibniz Institute for Age Research/Fritz Lipmann Institute, Jena) focused in his keynote lecture on the regulation of the GTP-binding protein p21-Ras and its role in determining cell fate. Whereas Ras can be activated *via *growth factor receptors and the adaptor proteins Grb2/SOS, signals can be relayed together with an ERM complex to F-Actin. The ERM complex consists of Ezrin, Radixin and Moesin. Importantly, Ezrin is also a binding partner for the cytoplasmic part of CD44v6, a splice variant of the proteoglycan-rich cell adhesion molecule CD44 which is implicated in cell activation signalling, invasion and tumour survival. Down-modulation of CD44 is required for the tumor suppressive activity of p53. Merlin can counteract the Ezrin-mediated activation of Ras in the ERM complex. and, thus, balances Ras-mediated downstream signalling, thereby possibly also contributing to tumour-suppression.

**Tereza Benesova **and colleagues (Institut für umweltmedizinische Forschung, Düsseldorf) demonstrated that, interestingly, the susceptibility of skin cells towards the infrared A part of natural sunlight is affected by the circadian rhythm. The central role of mitochondrial DNA in photoaging was underscored by **Bianca Schuermann **and colleagues (Düsseldorf), who presented evidence that increased levels of photo-inducible mtDNA deletions are functionally relevant for intensified matrix degradation and are therefore responsible for detrimental changes in photoaged skin. Aging is also one major risk factor of cardiovascular diseases and therefore it is important to understand vascular aging on a cellular level: Here, Buechner et al. found that mitochondrial Telomerase reverse transcriptase (TERT) has a protective role in mitochondria by strongly contributing to mtDNA integrity. TERT is associated with nuclear SHP-2, which acts as a negative regulator for nuclear export of TERT and reduces the formation of reactive oxygen species, as reported by **Sascha Jakob **and colleagues (Düsseldorf). Thus, nuclear SHP-2 activity seems to be a major player in the delay vascular aging processes.

A range of outstanding presentations was experienced by attendees of the Workshop on **Signalling In Immune Cells**. **Mark Philips **(New York University School of Medicine) kicked off the day with a keynote lecture on the compartmentalization of Ras signalling in T cells. Using a combination of CFP-tagged Ras molecules and a YFP-tagged Ras binding domain (RBD) that specifically targets active Ras, he nicely showed that in response to anti-CD3 stimulation alone, Ras activation occurred upon the Golgi, whereas in the presence of costimulation *via *the integrin LFA-1, Ras activation occurs at both the plasma membrane (PM) and Golgi. Additionally, he showed by using the C1 domain of Protein Kinase C fused to GFP as a reporter for diacylglycerol (DAG) production, that the mechanism by which ICAM contributed to DAG production requires phospholipase D2.

**Stefan Feske **(New York University School of Medicine) presented data from patients with T^+^B^+ ^severe combine immunodeficiency (SCID). Lymphocytes from these patients show only intracellular calcium flux, but no contribution from the extracellular calcium channels, which led to the discovery of the ORAI and STIM proteins. He also presented the characterization of single point mutants for ORAI that indicate the protein oligomerizes in the membrane to form an ion channel. Additionally, data were presented for the ER calcium sensors STIM1 and STIM2 suggesting that sustained calcium flux plays an important role in cytokine production and the development of regulatory T cells.

In a short talk by **Claudia Brandt **(Deutsches Rheumaforschungszentrum, Berlin) data on the benefit of low dose cyclosporin (CsA) therapy in patients with atopy were presented. This treatment reduced T cell receptor signalling and increased the number of regulatory T cells (CD4^+^Foxp3^+^). In investigating the mechanism, the group suggests that low TCR signalling leads to a low expression of TGIF, which in turn enhances the effect of TGF-β thereby promoting T_reg _development.

**Doris Urlaub **and colleagues (University of Heidelberg) used modelling to address the question of how an NK cell decides whether or not to kill a target cell. A simple model was created containing the minimal elements, an activating receptor that acts *via *a Src kinase to phosphorylate the substrate, Vav1, and an inhibitory receptor that acts *via *the phosphatase Shp-1 to dephosphorylate the substrate. A key assumption is that Vav1 plays a central role in the decision making process. Various parameters, such as receptor clustering, were investigated and compared to the experimental data. The resulting model has the potential to provide insights into the integration of positive and negative signals during lymphocyte activation.

**René Eulenfeld **(Technical University of Aachen) presented data on a novel mechanism regulating the recruitment Gab1 to the plasma membrane. Gab1 is an adapter, consisting of a single pleckstrin homology (PH) domain and a long 'tail' containing multiple tyrosine residues by which in recruits RasGAP, PI-3 kinase and Shp-2. In resting cells, Gab1 is cytosolic, but upon stimulation with cytokine (+IL-6) it translocates to the PM, where it becomes phosphorylated. Translocation is dependent on the PH domain and could be blocked by inhibiting PI-3 kinase activity. Interestingly, the MEK inhibitor U0126 also blocked membrane recruitment of Gab1. Further analysis identified the ERK-dependent phosphorylation of Ser551 as a key step regulating the translocation of Gab1. Thus it appears that Ser551 falls within an autoinhibitory domain that regulates the function of the PH domain.

Another keynote lecture was presented by **Ed Clark **(University of Washington) on the regulation of B cell entry into the cell cycle. Data was presented showing that the adapter Bam32 promotes entry into the cell cycle, while superoxide has the opposite effect. Both Bam32 and the NADPH oxidase complex compete for binding of PIP_2 _and Rac1, and by doing so may fine-tune signalling within B cells. Additional data were presented on the role of caspases in regulating the cell cycle. Interestingly, caspase 6-deficient B cells were prone to differentiate into plasma cells, rather than proliferate upon stimulation. This appears to be due to a dysregulation of the cell cycle regulator retinoblastoma (Rb).

**Facundo Batista **(London Research Institute) presented data on B cell responses to antigen. Membrane tethered antigens presented by either follicular dendritic cells (FDCs) or dendritic cells (DCs) appear to be of particular importance. Using the DT-40 B cell system, the influence of a number of signalling molecules on microcluster formation and cell spreading were investigated, as well as the role of the coreceptor, which appears to be important for antigens when they are membrane bound, but not in a soluble form.

**Jürgen Wienands **(University of Göttingen) investigated the signalling differences between naive and antigen-experienced B cells. He characterized a tyrosine-based signalling motif found within the cytoplasmic tail of the IgG heavy chain that upon stimulation becomes phosphorylated by Syk and recruits Grb2 to enhanced PLCγ activation and calcium flux. Thus, this motif appears to play a costimulatory-like function in B cell receptor signalling.

The DT-40 system was also used by **Edgar Serfling **and colleagues (University of Würzburg) who showed that high levels of NFATc1/alphaA-protected B cells from activation-induced cell death (AICD) by promoting the transcription of anti-apoptotic molecules such as Bcl-6. Since NFATc1/alphaA appears to control genes related to affinity maturation, its expression in germinal center (GC) B cells was investigated by immunohistochemistry. A strong nuclear localization was observed in a subset of GC B cells, which is in striking contrast to the cytosolic staining observed in most lymphocytes. Since this subset of cells appeared to express neither IgM nor apoptotic markers, it was proposed that NFATc1/alpha A contributes to the survival of mature GC B cells.

**Simone Keck **(University of Freiburg) presented data on the role of the Src kinase Lyn in negatively regulating Toll-like receptor (TLR) signalling in bone marrow-derived macrophages. Her data showed that macrophages from Lyn-deficient mice secreted higher levels of proinflammatory cytokines in response to TLR-ligands. She also observed a complex of Lyn and PI-3 kinase. To test if this was the mechanism of action, she inhibited PI-3 kinase with wortmannin and showed that cytokine production was increased. Additionally, in SHIP1-deficient mice, she observed a decrease in cytokine production in response to TLR-stimulation. Thus, she concluded that the negative regulatory function of Lyn upon cytokine production is at least in part mediated via PI-3 kinase.

The Workshop on **Adhesion, Motility, Morphology **started with a keynote lecture by **Harvey McMahon **(Medical Research Council, Cambridge) on the various mechanisms of endocytotic processes. He showed that endocytosis can occur in very different ways. McMahon especially distinguished endocytotic pathways that involve the canonical coat protein clathrin, or are independent of this protein, defined as pleiomorphic clathrin-independent carriers (CLICs) and GPI-enriched endocytic compartments (GEECs). Furthermore, he described the GTPase activating protein GRAF1 as a key regulator of the CLIC and GEEC endocytic pathways. The session continued with a contribution by **Cornelia Dietrich **from (University of Mainz). She presented data on the regulation and signalling pathways of the aryl hydrocarbon receptor (AhR), which is a transcription factor involved in physiological processes as well as toxic responses to 2,3,7,8-tetrachlorodibenzo-p-dioxin (TCDD). Treatment of rat liver oval cells led to induction of the transcription factor JunD, resulting in transcriptional upregulation of the proto-oncogene cyclin A which in turn led to a loss of contact inhibition and increased proliferation of the oval cells.

**Lars Müller **(University of Kiel) reported on the regulation of cancer-associated fibroblasts (CAFs) under the influence of the cytokine tumour necrosis factor (TNF)-alpha. He provided data that this inflammatory activation of the transcription factor NF-κB in CAFs leads to the release of interleukin-6, the monocyte-chemotactic protein-1 and other effects. The release of such inflammatory factors by CAFs in response to TNF-α promotes chemotaxis of colon carcinoma cells towards CAFs and might provide an explanation for colorectal metastasis formation in the liver.

**Sven Kröning **(University of Erlangen), introduced a novel cell migration assay. He explained artificial surfaces such as plastic bottoms of culture dishes as one major problem in the use of two-dimensional migration assay. He described a new system similar to the traditional scratch assay: In his experimental setup, the surface is coated with components of the extracellular matrix. A barrier is introduced in the cell culture, which is removed after the cell growth has reached confluency to provide a cell-free space corresponding to the scratch in the conventional scratch assay. The advantage of this procedure is due to the maintenances of the coated surface during the "scratch" process. The resulting gap is then closed in a quantifiable manner by migrating cells.

## Conclusion

The 2008 STS meeting clearly showed that signal transduction research has grown from its timid beginnings in Germany into a vibrant research endeavour that also attracts a growing number of international colleagues from many different research areas.

The 2009 conference will be held again in Weimar from 28 to 30 October. The special focus topic for 2009 will be 'The Molecular Biology and Signaling of Ageing'

## Competing interests

The authors declare that they have no competing interests.

## Authors' contributions

KF collected information from all other authors, organized and edited the contributions and drafted the manuscript. JAL, FE, ES, GT, AK, KG, CE, SMF, OU, FS, OJ and RH provided individual sections of text. All authors read and approved the final manuscript.

